# Pain and the Surgeon

**Published:** 1897-09

**Authors:** 


### SELECTIONS.

### Pain and the Surgeon.

Many surgeons study pain in a somatic more than they do in a psychic way. It is as essential to study the various phases of psychic as of bodily pain. Mental shocks in the weakened and suffering have debarred cure. It is not necessary to create a catastrophe to shock those mentally weak, but we venture to assert that the rough indifference of the surgeon and the display of instruments have, combined, killed more than the mere operation. Trivialities carefully studied lead to perfection. Picture, if you will, a weak, delicate, suffering woman, whose brain dwells in morbid sensibility. She comes to a surgeon for operative procedure. Our lordly man of the knife has not time to indulge in the ordinary amenities of life and examines his patient in orderly, business fashion, without the faintest show of sympathy or feeling and tells in the shortest possible way what has to be done, and honestly says that he cannot tell what the result may be. He has not time to soothe, placate or encourage. The time comes for the operation. The patient approaches the table in painful and nervous apprehension; nothing is said to encourage her. Chloroform is given without a soothing suggestion. Here is a mind that a mental shock can and will influence violently, and this mind goes under the influence of chloroform in a state where a morbid suggestion may be the means of causing grave results. It is a poor surgeon who cannot soothe and quiet a pained mind as well as a pained body. The surgeon is more often an unconscious inflicting agent, in operative work than he thinks.

A surgeon unacquainted with psychic pain is only half educated, and in truth the surgery of to-day becomes more mechanical and less soulful. Narrow, technique surgery makes most of its successes through the thoughts of other brains than that of the operator. If genius were required to make surgeons, then indeed they would be very few and far between. Technique is mechanical, not intellectual. The surgeon should not only be skilled in technique, but should have knowledge of every element of his art. He should be a student of the mind as well as of the body, and success only comes in its best form when we interpret every condition and surrounding of the patient aright.—*The Railway Surgeon.*

